# Fast reconstruction algorithm based on HMC sampling

**DOI:** 10.1038/s41598-023-45133-z

**Published:** 2023-10-18

**Authors:** Hang Lian, Jinchen Xu, Yu Zhu, Zhiqiang Fan, Yi Liu, Zheng Shan

**Affiliations:** Information Engineering University, Zhengzhou, China

**Keywords:** Mathematics and computing, Computer science, Information technology

## Abstract

In Noisy Intermediate-Scale Quantum (NISQ) era, the scarcity of qubit resources has prevented many quantum algorithms from being implemented on quantum devices. Circuit cutting technology has greatly alleviated this problem, which allows us to run larger quantum circuits on real quantum machines with currently limited qubit resources at the cost of additional classical overhead. However, the classical overhead of circuit cutting grows exponentially with the number of cuts and qubits, and the excessive postprocessing overhead makes it difficult to apply circuit cutting to large scale circuits. In this paper, we propose a fast reconstruction algorithm based on Hamiltonian Monte Carlo (HMC) sampling, which samples the high probability solutions by Hamiltonian dynamics from state space with dimension growing exponentially with qubit. Our algorithm avoids excessive computation when reconstructing the original circuit probability distribution, and greatly reduces the circuit cutting post-processing overhead. The improvement is crucial for expanding of circuit cutting to a larger scale on NISQ devices.

## Introduction

Quantum computing has demonstrated its superiority over classical computer in various scientific fields^1^, including machine learning^2,3^, chemistry^4,5^, code breaking^6^, finance^7,8^, and other fields^9^, where some quantum algorithms have exponential speedup in theory over classical algorithms. However, due to technical limitations, available quantum devices are currently called Noisy Intermediate-Scale Quantum devices. "Noisy" indicates that quantum devices are affected by noise, while "intermediate-scale" signifies that the number of qubits in these devices ranges from 50 to several hundred^10^. In the NISQ era, the challenge of constructing a reliable quantum device increases significantly as the number of qubits increases due to quantum noise. As a result, the current NISQ devices suffer from limited reliability and scalability^11^. The limitation of qubit resources leads to many quantum algorithms that cannot be applied to practical problems and do not reflect the superiority of quantum computing.

To address this problem, scholars worldwide have been studying circuit knitting techniques, which can simulate larger quantum circuits on small-scale quantum computing^12–18^. One of the crucial methods to realize circuit knitting is by implementing circuit cutting. Circuit cutting can divide large quantum circuits into multiple small quantum subcircuits, which can be executed independently. Moreover, the results of all subcircuits can be reconstructed into the theoretical results of the original circuit through additional classical post-processing algorithms. This approach enables us to run larger quantum circuits on a limited qubit resource quantum device, while incurring an increase in classical overhead costs.

Although circuit cutting is crucial to alleviate this problem of insufficient qubit resources in the NISQ era, circuit cutting currently has some limitations. The classical resources consumed by the post-processing of circuit cutting grow exponentially with the required number of cuts and the total number of qubits in the quantum circuit. Many algorithms have been developed to reduce post-processing overhead. For example, Lowe et al. addressed this issue by implementing random measurements^19^. Saleem et al. reduced the overhead by reducing the number of cuts^17^. Tang et al. proposed a dynamic-definition (DD) query algorithm, which can reduce the overhead by recursively and efficiently finding the circuit probability distribution from state space^18^. Chen et al. proposed an approximate reconstruction algorithm to sample the high probability solution space by MCMC sampling to reduce the overhead^20^.

This paper introduces a fast reconstruction algorithm. Through the evaluation of various random circuits, our reconstruction algorithm demonstrates an average runtime that is 45.6 times faster than the traditional exact reconstruction algorithm [Eq. (4)], 2.32 times faster than the DD algorithm, and approximately 1.47 times faster than the approximate reconstruction algorithm. Unlike the traditional exact reconstruction that reconstructs the probability distribution of the original circuit by traversing all possible quantum states, our reconstruction algorithm samples the high probability solutions by Hamiltonian dynamics from state space. By HMC sampling, our reconstruction algorithm reduces the overhead associated with reconstructing the original circuit results after each cut. Specifically, the contributions of this paper are as follows:

1. We propose a fast reconstruction algorithm based on HMC. The algorithm allows faster selection of high probability circuit results from state space by Hamiltonian dynamics, greatly reducing the time overhead.
2. In our reconstruction algorithm, only high probability solutions are sampled without the need for reconstructing all quantum states. This eliminates any 0-probability solution states or states with extremely low probabilities. The algorithm returns solution states with high probabilities, leading to a substantial reduction in space overhead.
3. We propose a method to decrease the number of cuts by decomposing two-qubit gates. This approach is employed in the experimental circuit described in this paper, which successfully reduces the number of cuts to 1.

This paper is organized as follows. In “Background”, we provide a brief introduction to the relevant fundamentals. In “Methods”, we outline the main content of our reconstruction algorithm. In “Results”, we conduct single cut reconstruction experiments using our reconstruction algorithm along with other approaches. In this section, a virtual two-qubit gate technique is employed to preprocess the reconfigured experimental circuit, enabling successful single cut splitting. In “Conclusion”, we conclude the paper and offers perspectives for future work.

## Background

### Circuit cutting

Circuit cutting is also known as a time-like cut. In quantum computing, any unitary matrix can be theoretically decomposed into a set of combinations of Pauli matrices $\{I,X,Y,Z\}$^18^. Specifically, any matrix A can be decomposed into the following formulas:

1 $$A=\frac{Tr(AI)I+Tr(AX)X+Tr(AY)Y+Tr(AZ)Z}{2}$$

We can further decompose the Pauli matrix into its eigenvalues and eigenvectors, and again derive the following Eq. (2)

2 $$\begin{aligned} & {A}_{1}=\left[Tr\left(AI\right)+Tr\left(AZ\right)\right]\left|0\right.\rangle \langle \left.0\right| \\ &{A}_{2}=\left[Tr\left(AI\right)-Tr\left(AZ\right)\right]\left|1\right.\rangle \langle \left.1\right| \\ &{A}_{3}=Tr(AX)[2\left|+\right.\rangle \langle \left.+\right|-\left|0\right.\rangle \langle \left.0\right|-\left|1\right.\rangle \langle \left.1\right|] \\ & {A}_{4}=Tr(AY)[2\left|+i\right.\rangle \langle \left.-i\right|-\left|0\right.\rangle \langle \left.0\right|-|\left|1\right.\rangle \langle \left.1\right|] \\ & A=({A}_{1}+{A}_{2}+{A}_{3}+{A}_{4})/2 \end{aligned}$$

**Figure 1:**
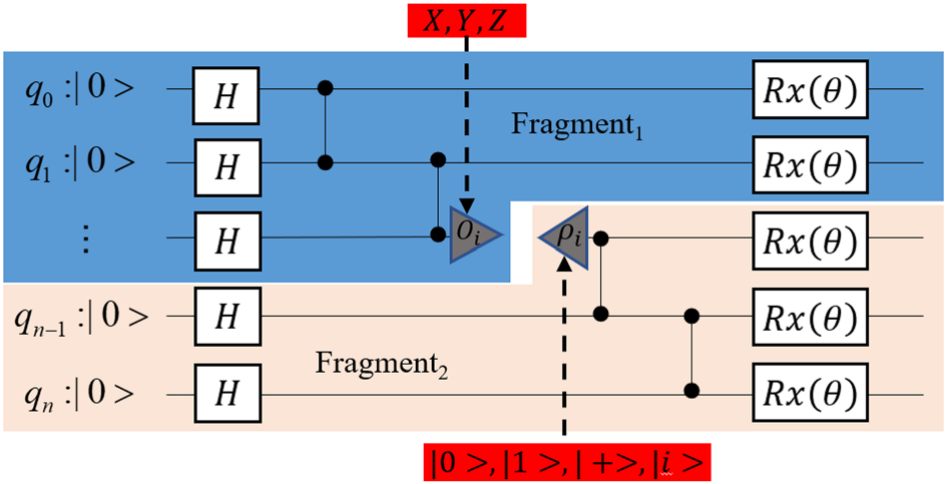
A quantum circuit can be divided into two parts using a single cut. These two parts create multiple subcircuits by incorporating different measurement bases and initialization operations. These subcircuits can run independently and the output of the original circuit can be reconstructed through classical post-processing.

Each trace operator corresponds to a set of measurements in a particular Pauli basis, while the density matrix comprising of eigenvectors corresponds to a set of initialization operations. Since the physical implementation of the measurement in the $I$ base and $Z$ base is identical, we can knit together the cut points by three measurement operations ($X,Y,Z$) and four initialization operations ($\left|0\right.\rangle ,\left|1\right.\rangle , \left|+\right.\rangle , \left|i\right.\rangle$). This facilitates the generation of three distinct upstream subcircuits (referred to as Fragment 1) with different measurement bases, and four downstream subcircuits (referred to as Fragment 2) with different initializations, as illustrated in Fig. 1. These subcircuits can be run independently, and the results can be measured.

However, it should be noted that since the last qubit of the upstream subcircuit does not appear in the final output of the uncut circuit, the results of the upstream subcircuits need additional processing. The results of the upstream subcircuits need to be multiplied by a factor $\alpha =\pm 1$. The sign of $\alpha$ depends on both the measurement base and the measurement result of the last qubit. When the measurement base is $I$, $\alpha =1$ regardless of the measurement result of the last qubit. However, when the measurement base is $\{X,Y,Z\}$, $\alpha =1$ if the measurement result of the last qubit is $\left|0\right.\rangle$, and $\alpha =-1$ if the measurement result is $\left|1\right.\rangle$.This relationship is summarized in Eq. (3) below:

3 $$\begin{aligned} &\overline{x0},\overline{x1}\to +\overline{x},M=I \\ & \overline{x0}\to +\overline{x},\\ & \overline{x1}\to -\overline{x},M=\{X,Y,Z\} \end{aligned}$$

Following the processing of the upstream subcircuit, it is necessary to compute the terms that correspond to both the upstream and downstream subcircuits to reconstruct the original circuit's probability distribution. Assuming that the original is cut as in Fig. 1, the cut position is at the $n/2$_nd_ qubit, and the circuit quantum state is $\left|{x}_{0}{x}_{1}{x}_{2}\dots {x}_{n}\right.\rangle$. The quantum state associated with the upstream subcircuit is ${x}_{up}=\left|{x}_{\left\{\frac{n}{2}+1\right\}}\dots {x}_{n}\right.\rangle$, and the quantum state associated with the downstream subcircuit is ${x}_{down}=\left|{x}_{0}\dots {x}_\frac{n}{2}\right.\rangle$ ,which ${x}_{i}\in \left\{\mathrm{0,1}\right\}$.

According to Eqs. (2) and (3), we know that during the reconstruction process, the upstream subcircuit consists of four terms,

$${p}_{\mathrm{1,1}}=p(|0{x}_{up}\rangle \mid I)+p(|1{x}_{up}\rangle \mid I)+p(|0{x}_{up}\rangle \mid Z)-p(|1{x}_{up}\rangle \mid Z)$$

$${p}_{\mathrm{1,2}}=p(|0{x}_{up}\rangle \mid I)+p(|1{x}_{up}\rangle \mid I)-p(|0{x}_{up}\rangle \mid Z)+p(|1{x}_{up}\rangle \mid Z)$$

$${p}_{\mathrm{1,3}}=p(|0{x}_{up}\rangle \mid X)-p(|1{x}_{up}\rangle \mid X)$$

$${p}_{\mathrm{1,4}}=p(|0{x}_{up}\rangle \mid Y)-p(|1{x}_{up}\rangle \mid Y)$$

The four terms of the downstream subcircuit are

$${p}_{\mathrm{2,1}}=p\left(|{x}_{down}\right.\rangle \left|\left|0\right.\rangle \right)$$

$${p}_{\mathrm{2,2}}=p\left(|{x}_{down}\right.\rangle \left|\left|1\right.\rangle \right)$$

$${p}_{\mathrm{2,3}}=2p\left(|{x}_{down}\right.\rangle \left|\left|+\right.\rangle \right)-p\left(|{x}_{down}\right.\rangle \left|\left|0\right.\rangle \right)-p\left(|{x}_{down}\right.\rangle \left|\left|1\right.\rangle \right)$$

$${p}_{\mathrm{2,4}}=2p(|{x}_{down}\rangle ||i\rangle )-p(|{x}_{down}\rangle ||0\rangle )-p(|{x}_{down}\rangle ||1\rangle )$$

The probability of the original circuit quantum state, denoted as $\left|{x}_{0}{x}_{1}{x}_{2}\dots {x}_{n}\right.\rangle$, can be calculated by taking the Kronecker product of the output terms from the upstream and downstream subcircuits. These output terms form 4 pairs, and their results are subsequently summed up, as shown in Eq. (4).

4 $$p(\left|{x}_{0}{x}_{1}{x}_{2}\dots {x}_{n}\right.\rangle )=p(\left|{x}_{down}{x}_{up}\right.\rangle )=\frac{1}{2}{\sum }_{i=1}^{4}{p}_{1,i}\otimes {p}_{2,i}$$

The probability distribution of the original circuit is obtained simply by calculating the probability of each quantum state in the original circuit using Equation (4).

### Virtual two-qubit gate

A virtual two-qubit gate is a technique that can decompose a two-qubit gate into a series of single-qubit gates, also known as a space-like cut. It essentially involves classical post-processing and sampling of single-qubit gates to ‘simulate’ the entanglement effect produced by two-qubit gates. This means that a two-qubit gate can be expressed as a series of individual quantum gate operations, such as the Pauli matrices $\{X, Y, Z\}$, as well as single-qubit rotating gates $Rx$,$Ry$, and $Rz$ around the $X$, $Y$, and $Z$ axes.

The super-operator $S\left(U\right)$ shown in Eq. (5) corresponds to the evolution of a complete quantum state, where $\rho$ is the density matrix of the quantum state and $U$ is the unitary operator acting on the quantum state,

5 $$S(U)=U\rho {U}^{\dagger}$$

Suppose ${A}_{1},{A}_{2}$ are both unitary operators, with

6 $$\begin{gathered} {\mathcal{S}}\left( {e^{{i\theta A_{1} \otimes A_{2} }} } \right) = \cos^{2} \theta {\mathcal{S}}(I \otimes I) + \sin^{2} \theta {\mathcal{S}}\left( {A_{1} \otimes A_{2} } \right) \hfill \\ + \frac{1}{8}\cos \theta \sin \theta \sum\limits_{{\left( {\alpha_{1} ,\alpha_{2} } \right) \in \{ \pm 1\}^{2} }} {\alpha_{1} } \alpha_{2} \left[ {{\mathcal{S}}\left( {\left( {I + \alpha_{1} A_{1} } \right) \otimes \left( {I + i\alpha_{2} A_{2} } \right)} \right)} \right. \hfill \\ \left. { + {\mathcal{S}}\left( {\left( {I + i\alpha_{1} A_{1} } \right) \otimes \left( {I + \alpha_{2} A_{2} } \right)} \right)} \right] \hfill \\ \end{gathered}$$

**Figure 2:**
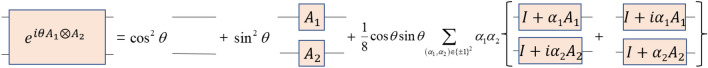
Decompose the two-qubit gate into a series of single-qubit gate sequences.

Therefore, it can be deduced that a two-qubit gate of form ${e}^{i\theta {A}_{1}\otimes {A}_{2}}$ can be decomposed into a series of single-qubit gates as shown in Fig. 2

If $A \in \{X,Y,Z\}$, then $I+{\alpha }_{1}{A}_{1}$ can be realized by projection measurements on the A-base, and $I+i{\alpha }_{2}{A}_{2}$ can be realized by a single qubit gate in rotation around the A-base, as derived in Ref.^21^.

### Hamiltonian Monte Carlo sampling

Hamiltonian Monte Carlo, also known as hybrid Monte Carlo^22^, is a Markov chain Monte Carlo (MCMC) sampling algorithm based on Hamiltonian dynamics. It describes the motion of a given object at time $t$ using its position $x$ and momentum $p$. Position corresponds to potential energy, while momentum corresponds to kinetic energy. Therefore, the potential energy $U(x)$ and kinetic energy $K(p)$ are functions of position and momentum, respectively. The Hamiltonian is the sum of the potential energy and kinetic energy functions. Thus, the Hamiltonian can be expressed as:

7 $$H\left(x,p\right) = U\left(x\right) + K\left(p\right)$$

The Hamiltonian characterizes the interconversion between kinetic energy and potential energy as an object moves. The following Hamiltonian equation can be analyzed quantitatively by differentiation.

8 $$\begin{array}{c}\frac{\partial {x}_{i}}{\partial t}=\frac{\partial H}{\partial {p}_{i}}=\frac{\partial K(p)}{\partial {p}_{i}}\\ \frac{\partial {p}_{i}}{\partial t}=-\frac{\partial H}{\partial {x}_{i}}=-\frac{\partial U(x)}{\partial {x}_{i}}\end{array}$$

If it is possible to express $\partial U(x)/\partial {x}_{i}$ and $\partial K(p)/\partial {p}_{i}$ with some initial conditions (e.g., at time ${t}_{0}$, initial position point ${x}_{0}$, and initial momentum ${p}_{0}$), it becomes feasible to predict the object's position and momentum at any subsequent time instant $t = {t}_{0}+ T$.

The Hamiltonian equation captures the continuous time evolution of an object's motion. To numerically simulate Hamiltonian dynamics, it is essential to discretize time to obtain an approximate solution for the Hamiltonian equation. The time interval $T$ can be divided into smaller sub-intervals of length $\delta$, allowing for an approximate continuity of time. This process can be accomplished using the leapfrog algorithm^23^, which sequentially updates the momentum and position variables. The algorithm proceeds by first calculating the momentum over a period, updating the object's position over a slightly extended period $\delta ,$ and finally completing the calculation of momentum for the next time interval. The algorithm follows the steps outlined below.

- Begin by calculating the change in momentum after half the time interval $\delta /2:$
  9 $${p}_{i}\left(t+\frac{\delta }{2}\right)={p}_{i}(t)-\frac{\delta }{2}\frac{\partial U}{\partial {x}_{i}(t)}$$
- Next, compute the change in position over the entire time interval $\delta$:
  10 $${x}_{i}(t+\delta )={x}_{i}(t)+\delta \frac{\partial x}{\partial {p}_{i}(t+\frac{\delta }{2})}$$
- Recalculate the change in momentum for the remaining half-time $\delta /2$:
  11 $${p}_{i}(t+\delta )={p}_{i}(t+\frac{\delta }{2})-\frac{\delta }{2}\frac{\partial }{\partial {x}_{i}(t+\delta )}$$

The core idea of HMC is to construct the Hamiltonian function $H(x,p)$. By leveraging this function, it becomes more efficient to explore the target distribution $P(x)$. The canonical distribution of statistical mechanics can be used to relate $H(x,p)$ to $P(x)$. Given the energy function of a state as $E(\theta )$, the corresponding canonical distribution can be defined as

12 $$P(\theta )=\frac{1}{Z}{e}^{-E(\theta )}$$

where Z is the regularization factor that ensures that $\int P(\theta )d\theta =1$, thus creating a valid probability distribution. Since the energy function is equal to the sum of potential and the kinetic energy in the system, it gives the following:

13 $$E(\theta )=H(x,p)=U(x)+K(p)$$

Then the canonical function of the Hamiltonian kinetic energy function can be expressed as

14 $$P\left(x,p\right)\propto {e}^{-H\left(x,p\right)}\propto {e}^{-U\left(x\right)}{e}^{-K\left(p\right)}\propto P\left(x\right)P\left(p\right)$$

According to Eq. (14), we can decompose the joint distribution $P(x,p)$ into the product of the distributions of $P(x)$ and $P(p)$, indicating that the two variables are independent of each other. As a result, their respective distributions can be utilized to sample their joint probability distributions. The introduction of an auxiliary variable $p$ can expedite the convergence of the Markov chain. Given that variables $x$ and $p$ are independent, the momentum variable $p$ can be sampled from any distribution, with $N(\mathrm{0,1})$ commonly being selected. The function connected to the potential energy in the Hamiltonian is given by:

15 $$K(p)=\frac{{p}^{T}p}{2}$$

In HMC, after defining $K(p)$, the remaining work is how to find the potential energy function $U(x)$ for a given target distribution $P(x)$, then the potential energy function is usually defined as:

16 $$U\left(x\right) = -logP\left(x\right)$$

Calculate again the gradient function $G(x)$ of the potential energy function:

17 $$G(x)=\frac{\partial U(x)}{\partial x}$$

Next, Hamiltonian dynamics can be applied to MCMC to sample the objective function $P(x)$. However, discretizing the time may introduce a specific error that may not match the target distribution, so the acceptance rate can be induced to offset the error, and the acceptance rate $\alpha$ is

18 $$\alpha =\mathit{min}\left(1,\mathit{exp}\left(-U\left({{\varvec{x}}}_{L}\right)+U\left({{\varvec{x}}}_{0}\right)-K\left({{\varvec{p}}}_{L}\right)+K\left({{\varvec{p}}}_{0}\right)\right)\right)$$

Here, $\left({x}_{0},{p}_{0}\right)$ represents the initial state, while the new state $\left({x}_{L},{p}_{L}\right)$ is obtained after executing the jump point algorithm $L$ times. A random point $u$ is chosen from a uniform distribution between 0 and 1. If the acceptance rate $\mathrm{\alpha }$ is greater than $u$, the point ${x}_{L}$ is accepted in the Markov chain. Upon performing multiple samples to reach the burn-in period of HMC sampling, the Markov chain converges towards a stationary distribution, which corresponds to the target distribution $P(x)$.

The random walk in the MCMC algorithm can lead the Markov chain to converge to a stationary distribution, $p(x)$, but it is often considered inefficient. Hamiltonian Monte Carlo leverages the principles of Hamiltonian dynamics in physics to calculate the future states of the Markov chain, rather than relying solely on a probability distribution. This approach enables more efficient exploration of the state space and achieves faster convergence compared to random wandering.

## Methods

### Fast reconstruction algorithm

Traditional exact reconstruction algorithms involve traversing through all possible quantum states in the original circuit's state space to reconstruct its probability distribution. This requires processing all potential combinations of qubit strings through brute force computation. However, the time complexity of these algorithms grows exponentially. As the number of qubits in the circuit increases, reconstruction time also experiences an exponential explosion. Additionally, exact reconstruction algorithms may encounter difficulties when applied to large-scale quantum circuits due to overhead. To overcome these challenges, this paper presents a fast reconstruction algorithm that differs from the approximate reconstruction method proposed by Chen et al. based on MH sampling^20^. In this work, we draw inspiration from MCMC sampling. However, our reconstruction algorithm deviates from the traditional MH approach, where the future state of a Markov chain is calculated through random wandering. Instead, our method employs Hamiltonian dynamics, rooted in the physical system concept, to determine future states. This technique enables more efficient analysis of the state space compared to random wandering and facilitates faster sampling of high probability solutions from state space, thus accelerating the convergence of Markov chains towards the original circuit's probability distribution. For a comprehensive understanding of HMC sampling, please refer to the references^24,25^.

Our reconstruction algorithm follows the steps outlined below.

- Step 1: Assume that the original circuit probability distribution is $P(x)$, then customize the potential energy function $U(x) = -logP(x)$, the gradient function $G\left(x\right)=\frac{\partial U(x)}{\partial x}$, and the kinetic energy function $K\left(p\right)=\frac{{p}^{T}p}{2}$.
- Step 2: Once the potential, gradient, and kinetic energy functions have been initialized, we must establish an initial state $\left[{x}_{0},{p}_{0}\right]$ for HMC sampling. Here, ${x}_{0}$ represents a randomly chosen quantum state from the original circuit's probability distribution, while ${p}_{0}$ typically denotes a random number drawn from a standard normal distribution $N(\mathrm{0,1})$. Additionally, it is essential to initialize a dictionary to store the original circuit's probability distribution.
- Step 3: Based on the HMC sampling principle mentioned above, simulating Hamiltonian dynamics in numerical terms requires discretizing continuous time. This is typically achieved using the leapfrog algorithm. To obtain the new state $\left[{x}_{l},{p}_{l}\right]$, the initial state $\left[{x}_{0},{p}_{0}\right]$ is iteratively updated L times using the leapfrog algorithm.
- Step 4: Once the new state has been obtained, it is necessary to calculate the acceptance rate $\mathrm{\alpha }=min\left(1,exp\left(-U\left({x}_{l}\right)+U\left({x}_{0}\right)-K\left({p}_{l}\right)+K\left({p}_{0}\right)\right)\right)$ for the state $\left[{x}_{l},{p}_{l}\right]$. This calculation is crucial because HMC sampling estimates the posterior distribution through probabilistic sampling. In essence, HMC sampling constructs a Markov chain that traverses the state space. The traversal is achieved by computing future states using the principles of dynamics in physical systems. To ensure that this Markov chain possesses the properties of a stationary distribution, it is necessary to define an acceptance rate $\mathrm{\alpha }$ for determining the viability of transitioning to future states. If the acceptance rate $\mathrm{\alpha }$ is greater than a threshold $u$, the point is accepted as a value from the original circuit's probability distribution and stored in the dictionary for multiple iterations. Following the burn-in period of the algorithm, the Markov chain produced by HMC sampling stabilizes into the desired stationary distribution, which corresponds to the original circuit's probability distribution.

The pseudocode for our reconstruction algorithm can be obtained based on the description above.

**Algorithm 1:**
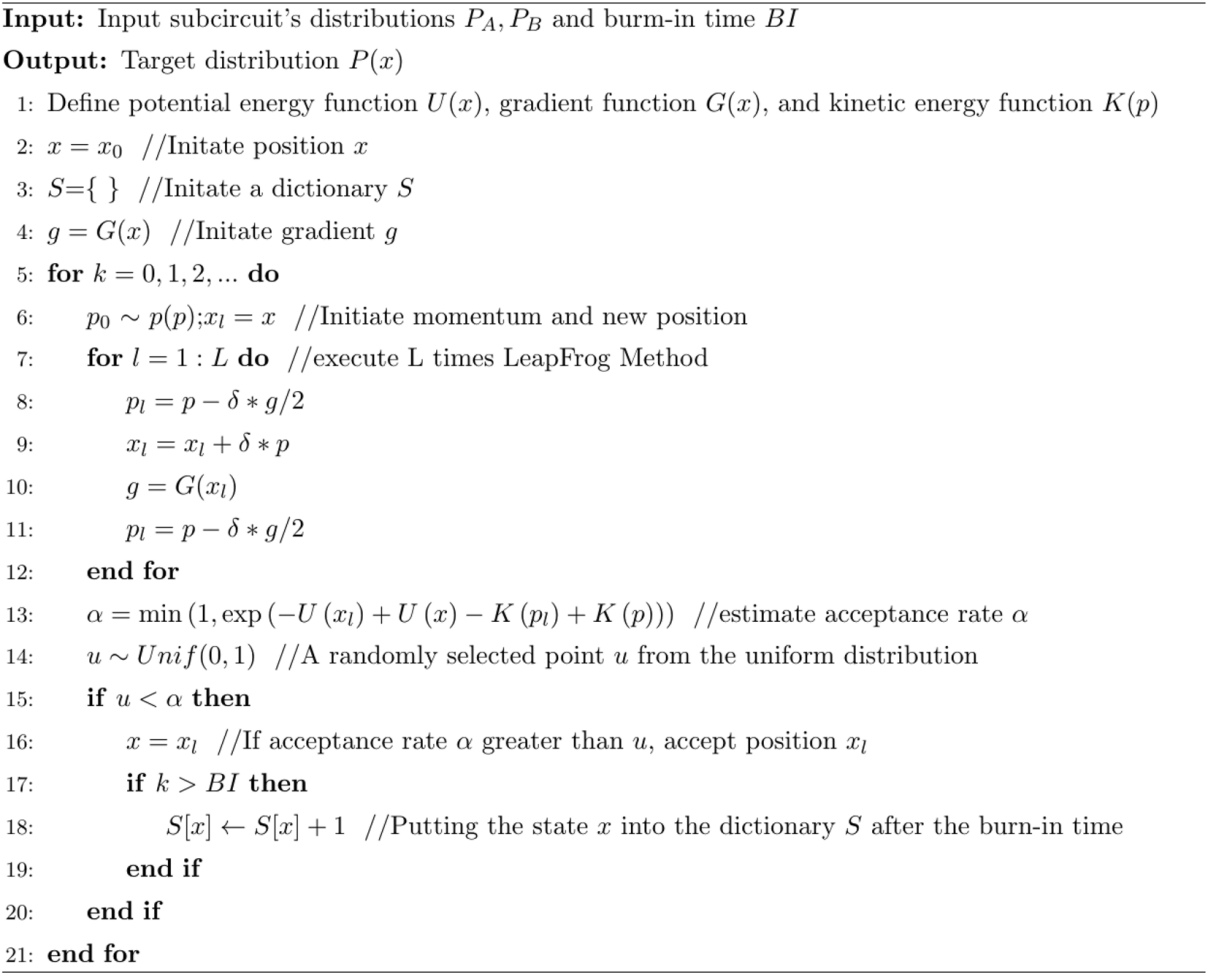
Fast reconstruction Algorithm.

## Results

As the overhead of circuit reconstruction grows exponentially with the number of cuts, this paper also investigates how to reduce the minimum number of cuts required for circuit cutting, which can be achieved by virtual two-qubit gate decomposition.

**Figure 3:**
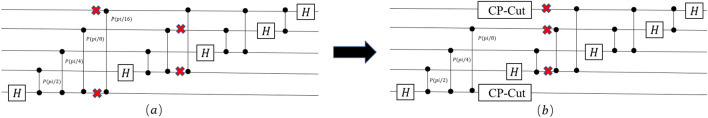
Comparison of the change in the minimum number of cuts required before and after two-qubit gate decomposition for a 5-qubit QFT circuit.

The two-qubit gate decomposition technique can be applied to certain fully connected quantum circuits to reduce their structural complexity, resulting in fewer cuts and a significant reduction in the circuit's processing overhead. Figure 3 illustrates the change in the minimum number of required cuts before and after applying a two-qubit gate decomposition on a fully connected 5-qubit QFT^26^ circuit.

If the circuit shown in Fig. 3a is cut directly, we need to cut it at least 4 times to decompose the circuit. We try to decompose the CP gate between qubit 0 and qubit 3 of the 5-qubit QFT circuit, the decomposition method is shown in Equation (6), and the decomposed circuit is shown in Fig. 3b. The CP-Cut in Fig. 3b indicates the gates after the CP gate is decomposed.

**Figure 4:**
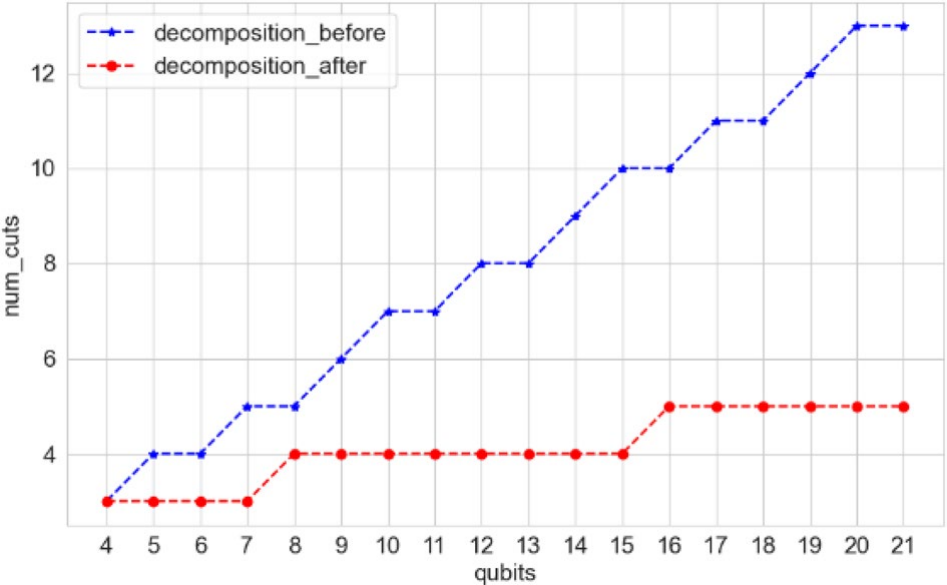
Comparison of the minimum number of cuts required for QFT circuit before and after decomposing a two-qubit gate.

After applying the two-qubit decomposition principle to decompose the 5-qubit QFT circuit, it was found that only three cuts were needed to separate the circuit. Furthermore, we conducted additional experiments using the circuit-knitting-toolbox toolkit to explore the furthest two-qubit gate decomposition for varying qubit sizes in QFT circuits. The minimum number of cuts required before and after decomposing the two-qubit gate was then calculated. The experimental results are depicted in Fig. 4.

**Figure 5:**
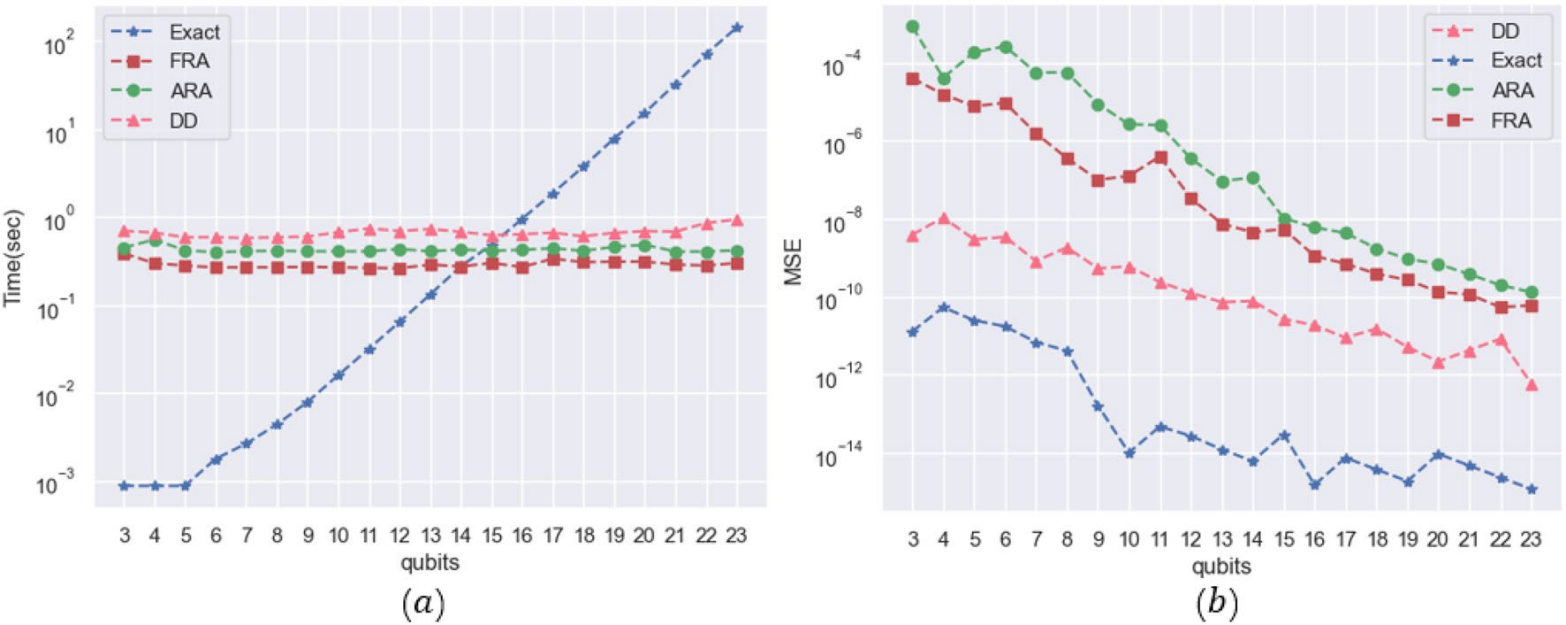
Comparison of runtime and $MSE$ for different post-processing algorithms for a single cut of random circuits.

Analysis of Fig. 4 reveals that decomposing two-qubit gates effectively reduces the number of cuts in 4-qubit to 21-qubit QFT circuits. This reduction is more prominent as the number of qubits in the QFT circuit increases. This trend can be extrapolated to complex quantum circuits, where decomposition of two-qubit gates serves as a strategy to minimize circuit cuts. Larger circuits benefit more from this technique, experiencing a greater reduction in the number of cuts after the decomposition of two-qubit gates. This paper evaluates the performance of our reconstruction algorithm through several experiments. Specifically, we measure the runtime of single cut reconstruction for randomized circuits with varying qubit sizes and compare it against three other algorithms: the traditional exact reconstruction algorithm, Tang's DD algorithm, and Chen's approximate reconstruction algorithm. In cases where a single cut is insufficient to cut the circuit, we apply two-qubit gate decomposition to minimize the number of required cuts. These experiments were conducted using the qasm simulator, and the corresponding results are illustrated in Fig. 5a.

Figure 5a shows that the traditional exact reconstruction algorithm (Exact) shows remarkably short runtime for small qubit sizes. However, due to its requirement to traverse all quantum states, the runtime of Exact experiences exponential growth as the number of qubits in the quantum circuit increases. Conversely, the other three reconstruction algorithms demonstrate smoother time distributions in Fig. 5a and do not show significant fluctuations with increasing qubit counts. Among the three algorithms, the DD algorithm shows the longest average runtime due to its utilization of continuous recursion for merging active qubits into $bins$, aiming to reconstruct solution states^18^. However, the time-consuming process of merging quantum states per recursion contributes to the relatively slower average runtime of the DD algorithm compared to both the fast reconstruction algorithm (FRA) and the approximate reconstruction algorithm (ARA) based on sampling. Notably, FRA outperforms ARA in terms of speed as it incorporates the concept of Hamiltonian dynamics in physical systems to compute future states within the Markov chain, in contrast to the random wandering approach employed by ARA.

The experimental results indicate that FRA outperforms other reconstruction algorithms in terms of runtime. Specifically, FRA's average runtime is 45.6 times faster than the traditional reconstruction algorithm, 2.32 times faster than the DD algorithm, and around 1.47 times faster than ARA.

In addition to the runtime analysis, this paper also compares the correctness of all quantum states of the probability distribution of the reconstruction results, and the evaluation metric used in this paper is the mean squared error ($MSE$), as in Eq. (19):

19 $$MSE={\sum }_{i}{\left({x}_{i}- {y}_{i}\right)}^{2}$$

Here, ${x}_{i}$ refers to the reconstructed result, while ${y}_{i}$ represents the result obtained from original circuit. A smaller $MSE$ corresponds to a lower error rate, as depicted in Fig. 5b, which presents a comparison of the $MSE$ values for different algorithms. Based on the experimental findings, the Exact shows the lowest error rate and closest proximity to the probability distribution of the original circuit. It is followed by the DD algorithm, the FRA proposed in this paper, and finally ARA.

The $MSE$ of FRA shows improvement compared to the $MSE$ of ARA in this experiment. However, there exists a disparity between traditional exact reconstruction and DD algorithms. This discrepancy arises due to the inherent sampling-based nature of FRA and ARA, which introduces a certain degree of error in contrast to alternative algorithms.

Quantum circuits can be broadly classified into two types. The first type of circuit results in a sparse probability distribution, where only a few solution states have a significantly high probability, while non-solution states have a probability of 0. Quantum algorithms like QAOA^27^, Grover^28^, and BV^29^ generally belong to this type. On the other hand, the second type of circuit produces a dense probability distribution, where many quantum states have non-zero probabilities, such as the 2-D random circuits^30^. In QAOA and similar algorithms, it is sufficient to highlight the solution state in the reconstructed probability distribution to obtain the algorithm's solution. There is no need to excessively focus on achieving high accuracy for all possible solutions. Consequently, we have tested the first type of circuit, specifically the QAOA circuit, as demonstrated below using an example to address the Max-Cut problem.

**Figure 6:**
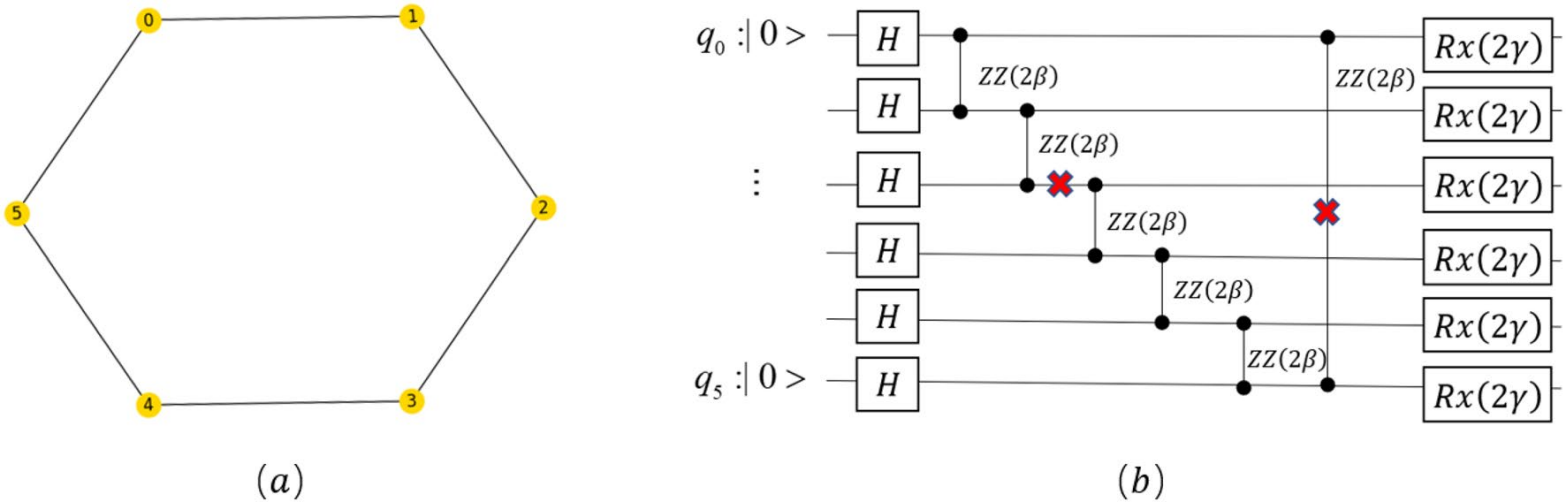
(**a**) is a diagram of the maximum cut problem to be solved, and (**b**) is a QAOA quantum circuit built by the objective function; we decompose the farthest CZ gate of the circuit and make one cut of the circuit.

This paper makes a single cut to the 6-qubit QAOA circuit. This circuit is used to solve the Max-Cut problem of Fig. 6a, and the probability distribution reconstructed using FRA in this paper is experimentally compared with the results of run of the original circuit.

The Max-Cut problem is a common combinatorial optimization problem in graph theory with important applications in statistical physics and circuit design. Michel Goemins and David Williamson proposed a classical algorithm based on semidefinite programming (SDP) approximation for solving the Max-Cut Problem in 1995, which is the best-known approximation algorithm for polynomial time^31^. The effectiveness of QAOA depends on the number of layers of the unitary transformation used, and in theory, it is possible to find an excellent approximation with enough layers, but it can also be time-consuming.

The Max-Cut problem involves dividing the nodes of a graph into two sets so that the number of edges between the sets is maximized. The objective function for its transformation into a combinatorial optimization problem can be formulated as follows:

20 $$C=\frac{1}{2}{\sum }_{ij\in E}\left({Z}_{i}{Z}_{j}-I\right)$$

Assuming a max-cut is performed on the graph shown in Fig. 6a, we can then construct a quantum circuit graph illustrated in Fig. 6b based on the objective function of the Max-cut, represented by Eq. (20).

**Figure 7:**
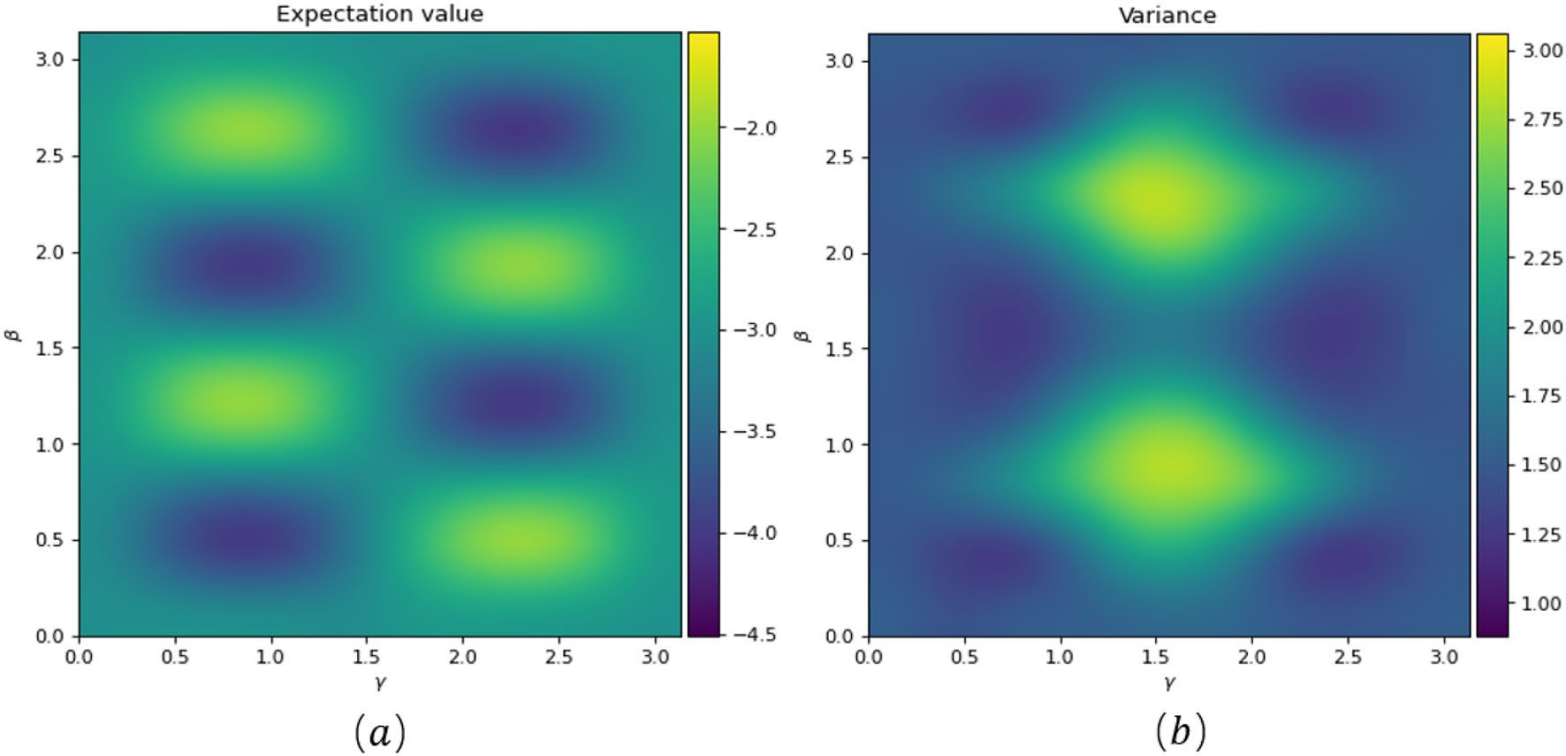
Figures (**a**) and (**b**) illustrate the expected value of the QAOA circuit and the variance of the measurements with the parameter $\upbeta$, $\upgamma$ for $p$ = 1 (where $p$ represents the number of iterations of the QAOA algorithm), respectively.

We optimize the parameters $\beta ,\gamma$ ($2\beta$ is the rotation angle of the $Rzz$ gate, $2\gamma$ is the rotation angle of the $Rx$ gate) by the classical $COBYLA$ optimizer. The changes in the circuit's expected value and variance are observed and presented in Fig. 7. In the expectation value measurement, a larger shaded region indicates a smaller value, indicating a better fit of the parameters and closer proximity of the circuit results to the approximate optimal solution. On the other hand, the variance represents the stability of the solution, where a larger shaded region corresponds to a smaller variance, indicating a more stable and reliable solution.

After finding the optimal parameters to construct the complete QAOA circuit in conjunction with Fig. 7, the farthest two-qubit gate $Rzz$ gate of the QAOA circuit is decomposed due to

21 $$Rzz(\theta )={e}^{-i\frac{\theta }{2}Z\otimes Z}$$

**Figure 8:**
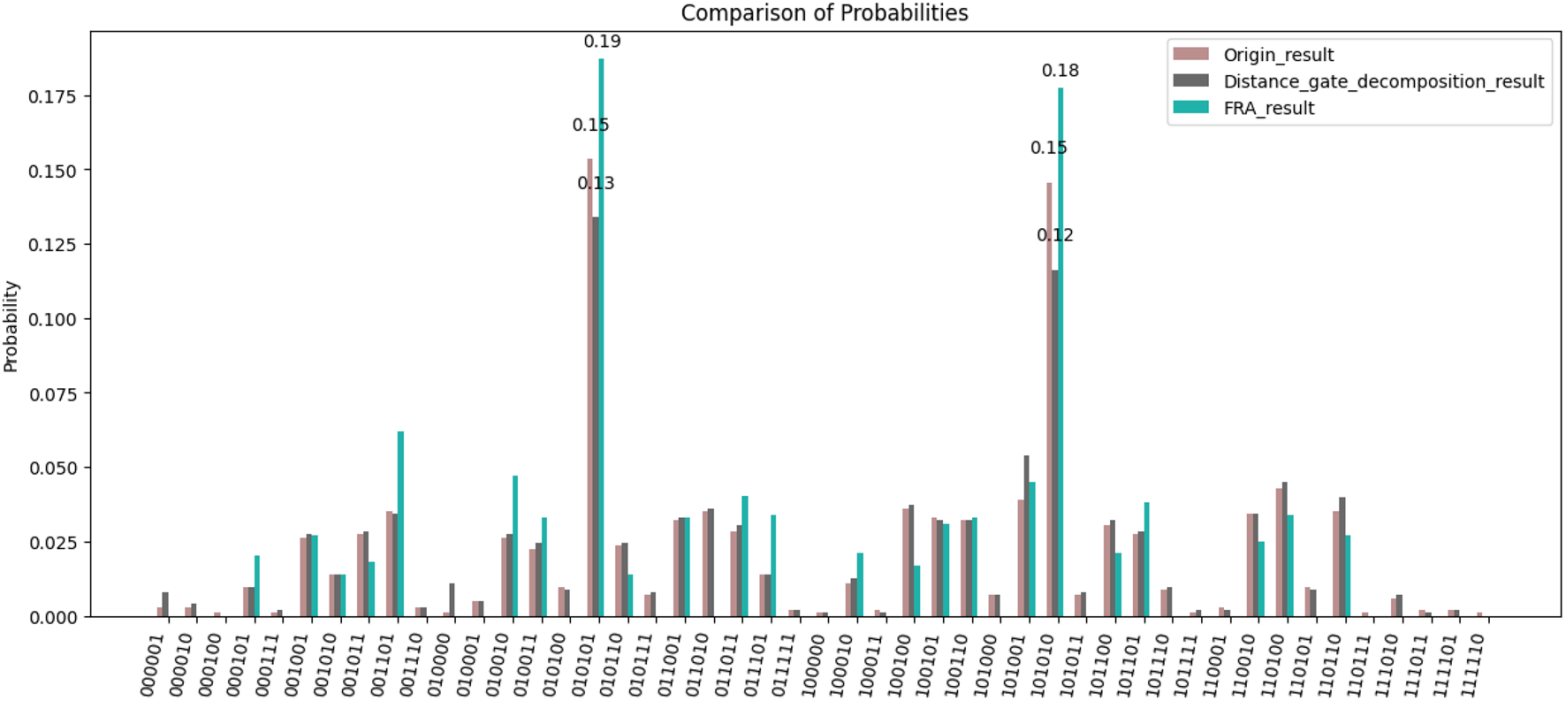
Results of the original circuit, the circuit after two-qubit gate decomposition, and results of running FRA on a single cut circuit after decomposing the two-qubit gate, with the probability values of some high probability solution states also marked in figure.

According to Eq. (6), the $Rzz$ gate can be decomposed into a series of $Z$ gates and a combination of $Rz$ gates. Subsequently, we divide the circuit into two parts by making a single cut and then reconstruct the circuit results using FRA. We conducted a comparative analysis of the results obtained from running the original circuit, the circuit after decomposing the two-qubit gate, and the reconstructed circuit after decomposing the two-qubit gate and performing a single cut. These results are visually represented in Fig. 8.

From Fig. 8, we see that the correct results of the circuit are |010101> and |101010>, and the correct solution probabilities of the circuit after decomposing the two-qubit gate are 0.13 and 0.12 respectively, which are lower than that of the original circuit at 0.15. However, the fast reconstruction algorithm is also able to reconstruct the high probability solution state based on the circuit after the two-qubit gate decomposition, with a correct solution probability of 0.19 and 0.18, which is even higher than that of the original circuit of 0.15. Although FRA does not fully reconstruct all quantum states within the state space of the original circuit, such as the low-probability solutions |011111> and others, this limitation results in a relatively low $MSE$. However, FRA shows stability in reconstructing high probability solutions, which closely resemble the original distribution. This level of accuracy is sufficient for solving circuits like QAOA.

To further validate the conclusion, we performed additional experiments on multiple QAOA circuits. Each circuit consisted of two high probability solutions, which were then evaluated through FRA after a single cut. The experiment results were sorted in descending order of probability after running all circuits. Subsequently, the first two solutions from the original circuits' running results were compared to the first two solutions of the FRA reconstruction results. The evaluation metric used in this comparison was the coincidence rate ($CR$).

22 $$CR=\left\{\begin{array}{ll}0,\hspace{0.33em}& \hspace{0.33em}no\hspace{0.33em}coincident\hspace{0.33em}solutions\hspace{0.33em}\\ 0.5,\hspace{0.33em}& \hspace{0.33em}one\hspace{0.33em}solution\hspace{0.33em}is\hspace{0.33em}coincident\\ 1,\hspace{0.33em}& \hspace{0.33em}the\hspace{0.33em}first\hspace{0.33em}two\hspace{0.33em}solutions\hspace{0.33em}are\hspace{0.33em}coincident\end{array}\right.$$

The $CR$ is equal to 1 when both first two solutions coincide completely, 0.5 when only one solution coincides, and 0 when there are no coinciding solutions.

**Figure 9:**
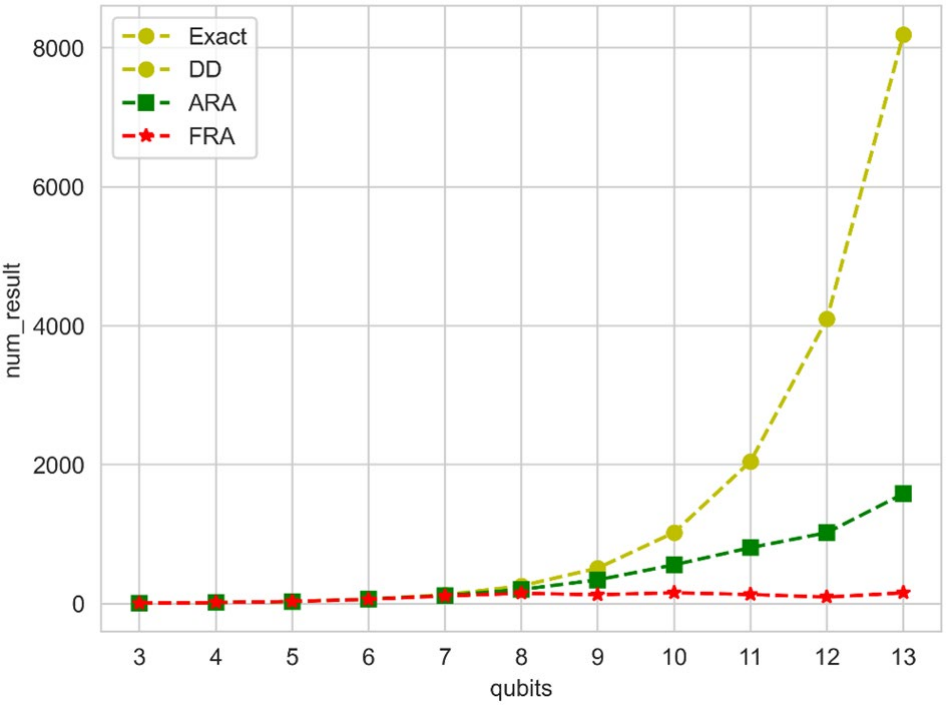
The Figure shows the number of reconstructed results obtained through various algorithms, namely Exact, DD, ARA, and FRA, for the 3-qubit to 13-qubit qubits QAOA experiment.

We conducted experiments on the QAOA circuits with 3-qubit to 13-qubit. As a result of the experiments, the $CR$ of the reconstructed results of the FRA for all experimental circuits is 1, when compared to the results obtained from the original circuits. This signifies that FRA is capable of accurately reconstructing the solution states for quantum circuits, such as QAOA. Additionally, we have counted the number of results obtained by various reconstruction algorithms in the experiments to estimate the space overhead, as depicted in Fig. 9.

As shown in Fig. 9, of all the reconstruction algorithms for this experiment, the FRA reconstruction yields the least number of solutions. The Exact and DD algorithms reconstruct all quantum states within the state space of the circuit. However, the results obtained by these algorithms increase exponentially as the number of qubits grows. At a certain point, the storage device may be incapable of accommodating all the results. Conversely, FRA reconstructs high probability solutions from the original circuit, eliminating the presence of 0-probability or low-probability solutions. Consequently, the storage space overhead can be significantly reduced, transforming from exponential to polynomial scale when compared to Exact and DD algorithm for large-scale quantum circuits.

In summary, FRA can efficiently acquire the reconstruction probability distribution of a quantum circuit in a shorter runtime compared to the Exact, DD, and ARA. Unlike the Exact and DD algorithm, FRA does not require the reconstruction of probabilities for all quantum states. Instead, it selectively focuses on high probability solutions, reducing time overhead and space overhead while finding the correct solution for the circuit. This means that FRA is a low overhead circuit-cutting post-processing algorithm.

## Conclusion

This paper introduces an algorithm based on Hamiltonian Monte Carlo in circuit cutting reconstruction. Additionally, we apply two-qubit gate decomposition to minimize the number of cuts in experimental circuits during our experiments. Our comprehensive experiments demonstrate that our reconstruction algorithm efficiently samples high probability solutions from state space, without the need to traverse all possible states. Furthermore, it significantly reduces both time and space overheads.

In the NISQ era, circuit cutting plays a crucial role in extending NISQ devices to larger scales. However, due to its excessive post-processing overhead, operating on large-scale quantum circuits becomes impractical. Therefore, the algorithm proposed in this paper to reduce the post-processing overhead of circuit cutting holds the utmost importance for the NISQ era. Additionally, the concept of the fast reconstruction algorithm, introduced in this paper, solely obtaining high probability solutions carries significant implications for the future development of quantum computing.

Currently, our reconstruction algorithm is only applicable to circuits with a single cut, and it has not been extended to circuits with multiple cuts. Our reconstruction algorithm is particularly suitable for circuits such as QAOA. Investigating and exploring how the algorithm can be adapted for multiple cuts and extended to all quantum circuits are promising directions for future research.

## Data Availability

The data presented in this paper is available online at https://github.com/hang-dev/FRA.

## Code availability

The code for the specific implementation of our method is available online at https://github.com/hang-dev/FRA.
